# Novel methods for health intervention research

**DOI:** 10.25646/6503

**Published:** 2020-06-04

**Authors:** Till Bärnighausen

**Affiliations:** University of Heidelberg Faculty of Medicine, Heidelberg Institute of Global Health

Research is important for the ideation, design, and testing of health interventions. Intervention research can be classified into four dimensions:

1. Identification of health intervention needs, which requires large-scale population-representative studies (e.g. [1–3]).
2. Design research to create interventions that are desirable, feasible and viable, which requires ethnographic studies, ideation, and prototype and pilot testing (e.g. [4–7]).
3. Evaluation research to quantify (i) causal effects and impacts – causal impact evaluation – [8–10], (ii) mechanisms of action – performance or process evaluation – [11], and (iii) social value of health interventions – economic evaluation [12].
4. Governance and policy translation research to guarantee the ethical and political ‘goodness’ of our approaches to real-life health intervention research [13, 14] and to ensure rapid incorporation of novel insights into health policy and routine practice [15].

For each of these four dimensions of health intervention research, I present several novel methodological approaches and illustrate them with examples from real-life studies in resource-poor communities in Africa and Asia. My focus is on innovative study designs: new forms of design research for health interventions [16], a range of innovative experimental and quasi-experimental approaches for causal impact evaluation [17–21], new methods to validly measure health and behavioral outcomes [22–24], novel approaches to quantify economic evaluation [25], and new methods for policy and public engagement. For each method, I explain the intuition, describe epistemic and statistical backgrounds, and discuss application opportunities, strengths and weaknesses.
